# Comparative Analysis of Clinical, Dermoscopic, and Confocal Microscopy Scores for Assessing Severity of Actinic Keratosis

**DOI:** 10.3390/cancers17172899

**Published:** 2025-09-03

**Authors:** Cristina Soare, Elena Codruța Cozma, Călin Giurcăneanu, Vlad Mihai Voiculescu

**Affiliations:** 1 Department of Dermato-Oncology, Faculty of Medicine, “Carol Davila” University of Medicine and Pharmacy, 050474 Bucharest, Romania; cristina.vajaitu@drd.umfcd.ro (C.S.); calin.giurcaneanu@umfcd.ro (C.G.); vlad.voiculescu@umfcd.ro (V.M.V.); 2 Department of Dermatology, “Elias” University Emergency Hospital, 011461 Bucharest, Romania

**Keywords:** actinic keratosis, dermoscopy, reflectance confocal microscopy, non-invasive diagnosis

## Abstract

*Background*: Actinic keratosis (AK) is a common UV-induced intraepidermal neoplasm and a precursor to cutaneous squamous cell carcinoma (cSCC). Accurate, non-invasive assessment of AK severity is crucial for early intervention and risk stratification. Objective: To evaluate the relationship between clinical severity of AK, as defined by the Olsen grading scale, and predefined dermoscopic and reflectance confocal microscopy (RCM) features. *Methods*: This cross-sectional study enrolled 50 patients with clinically diagnosed AK at a tertiary dermatology center between April 2024 and July 2025. Each lesion was assessed clinically using the Olsen scale (grades 1–3), dermoscopically (five features scored 0–3; total score 0–15), and via RCM (five RCM parameters scored 0–3; total score 0–15). Statistical correlations between clinical grade and imaging features were analyzed using Pearson’s χ^2^ test with effect size metrics. *Results*: Diffuse erythema (*p* < 0.001), micro-erosions (*p* = 0.002), strawberry pattern (*p* = 0.038), and scales (*p* = 0.012) correlated significantly with Olsen grade, whereas vessels (*p* = 0.566) did not. All five RCM parameters showed strong associations with clinical severity. Composite dermoscopic and RCM scores correlated with Olsen grade (both *p* < 0.001). Abnormal honeycomb pattern, parakeratosis, inflammation, and solar elastosis were the best RCM predictors of high dermoscopic severity (all *p* < 0.001); conversely, erosions, erythema, and scales were the strongest dermoscopic predictors of high RCM severity (all *p* < 0.001). *Conclusions*: This study demonstrates strong, statistically significant associations between clinical, dermoscopic, and confocal features of AK. This supports the integration of multimodal scoring into unified AK severity frameworks.

## Simple Summary

Actinic keratoses are common, UV-induced precancerous lesions. The possibility of progression to squamous cell carcinomas, as well as the impossibility of predicting which of these lesions will progress, necessitates dermatological evaluation and treatment of all actinic keratoses lesions. The present study aims to evaluate 50 actinic keratosis lesions clinically, by dermatoscopy and confocal reflectance microscopy, in order to identify prognostic scores and clinical–imaging correlations that allow for better diagnosis, classification, and monitoring of the efficacy of topical treatments for these lesions.

## 1. Introduction

Actinic keratosis (AK) is among the most prevalent precancerous lesions worldwide. However, due to its low mortality rate, these lesions are frequently neglected, leading to an increased risk of progression to cutaneous squamous cell carcinoma (cSCC). Long-term observational studies estimate that 0.025–16% of AKs evolve into cSCC and that 40–60% of cSCCs arise from pre-existing, untreated AKs [1,2,3].

AK is described as an intraepithelial keratinocytic dysplasia, clinically presenting as small, irregular, rough, pink, red, or beige skin lesions on sun-exposed areas of the body, including the face, ears, dorsal hands, and arms. When affecting the lips, it is termed actinic cheilitis (AC). Additionally, AK may manifest as verrucous hypertrophic lesions with thick scales on the surface, also known as hypertrophic actinic keratosis (HAC) [4,5]. Although traditionally regarded as a precancerous lesion, some authors consider AK an in situ neoplasm, given its clonal DNA mutations in keratinocytes [6,7,8,9,10]. Indeed, AKs exhibit malignant features from their onset, with cellular alterations in epidermal keratinocytes that closely resemble those of cSCC. These changes include loss of polarity, nuclear pleomorphism, dysregulated maturation, increased mitotic figures, and molecular alterations such as p53 mutations [11,12,13,14,15].

Several risk factors contribute to AK development, with chronic exposure to ultraviolet (UV) radiation from sunlight or indoor tanning being the most significant. Additional factors include geographic location (with higher prevalence near the equator), immunosuppression due to medical conditions or medications, genodermatoses associated with defects in DNA repair, lighter Fitzpatrick skin phototypes, and age over 40 years [16]. Reactive oxygen species (ROS), including singlet oxygen, play a dual role in AKs, both in its pathogenesis and as a therapeutic target. Chronic UV exposure promotes ROS generation in epidermal keratinocytes and dermal fibroblasts, leading to oxidative DNA damage, lipid peroxidation, and activation of signaling pathways that drive keratinocyte dysplasia and malignant transformation toward cSCC [17]. Singlet oxygen, in particular, has been identified as a key mediator of photo-oxidative injury in skin cells, influencing not only DNA integrity but also protein and lipid stability [18]. These oxidative processes also affect dermal papilla cells and melanocytes, with responses modulated by cell–cell interactions and local oxygen levels. This mechanistic overlap between ROS-driven damage in AK development and ROS-mediated cytotoxicity in PDT underscores the relevance of oxidative stress biology in both prevention and treatment strategies.

AK often does not present as a single lesion; rather, it commonly affects an entire field of chronically sun-exposed skin, a phenomenon termed “field cancerization” [19,20,21,22,23]. This includes both clinically visible AKs and subclinical lesions. Field-directed therapy is often necessary to effectively manage the entire affected area [24,25,26].

While histopathological examination remains the gold standard for confirmation in clinically atypical or suspicious cases, the diagnosis of AK is most often established on the basis of clinical examination. Diagnostic tools such as dermoscopy, RCM, and optical coherence tomography (OCT) can support clinical diagnosis, especially in ambiguous cases [27]. Histopathological confirmation is not routinely required if clinical findings are characteristic, given the impracticality and discomfort of performing biopsies across large areas of sun-exposed skin [28]. However, lesions with atypical features, signs of progression to cSCC, or those unresponsive to adequate therapy should undergo biopsy. Early diagnosis and treatment of AK can help prevent progression and facilitate the early detection of malignant transformation, thereby reducing the risk of subsequent complications [14].

Clinical severity is traditionally graded using the Olsen score, which classifies lesions based on thickness and palpability. However, advances in non-invasive imaging, including dermoscopy and RCM, offer the potential to enhance diagnostic accuracy and risk stratification [29]. The clinical grading system proposed by Olsen et al. classifies AK into three grades: Grade 1 (slightly visible but palpable), Grade 2 (visible and palpable), and Grade 3 (frankly visible and hyperkeratotic) [29,30].

## 2. Non-Invasive Diagnosis of Actinic Keratosis

Clinical assessment alone cannot always differentiate AK from cSCC in situ or invasive cSCC, with diagnostic accuracy ranging between 74% and 94% [10,31,32]. Dermoscopy allows visualization of subsurface vascular and keratin features, while RCM provides quasi-histological assessment of cellular and architectural changes. However, the relationships between these imaging modalities and clinical severity have not been fully elucidated [33]. Dermoscopy provides valuable insights into the clinical evaluation of AK, offering enhanced visualization of subsurface features that improve diagnostic accuracy. Several characteristic dermoscopic criteria have been described in the literature and are commonly employed to identify AK and differentiate it from other cutaneous lesions [33,34].

One of the most frequently observed dermoscopic features of AK is the presence of fine, wavy vessels, representing superficial vascular proliferation due to chronic UV-induced damage. Scale, typically manifesting as white or yellowish keratin flakes, is another consistent finding reflecting hyperkeratosis and parakeratosis [34]. Additional criteria include the rosette sign, consisting of four white points arranged in a rosette-like configuration under polarized light, often seen in follicular openings and associated with hyperkeratosis. Microerosions, small focal erosions within the lesion, may also be present and are indicative of superficial epidermal damage [35]. The strawberry pattern is a particularly distinctive feature of AK, characterized by an erythematous background with prominent follicular openings surrounded by a white-to-yellowish halo, resembling the surface of a strawberry. White structureless clods and white globules represent areas of compact keratin accumulation and correlate with hyperkeratosis [36,37,38].

Other dermoscopic signs that may be observed include the jelly sign (translucent appearance in some cases), rhomboidal pattern (intersecting lines forming rhomboid shapes, especially on sun-damaged skin), and the starburst pattern (radiating lines or streaks at the periphery). Inner gray halos and annular granular patterns (ring-like granularity) further contribute to the dermoscopic diagnosis of AK [37,38,39]. White circles and double white clods are additional features indicating keratin-filled follicular openings, while fingerprinting describes parallel, curvilinear lines resembling a fingerprint pattern. Central crusting may be seen in lesions with superficial erosion or ulceration, and grayish areas can appear as diffuse or focal pigmentation within the lesion, reflecting melanin deposition or pigment incontinence [38,39,40].

Collectively, these dermoscopic criteria enhance the diagnostic accuracy for AK, guiding clinicians in differentiating it from other non-melanocytic and melanocytic lesions and facilitating early intervention [40].

RCM offers higher diagnostic precision than clinical and dermoscopic assessment. RCM is a non-invasive, in vivo imaging modality that provides horizontal, cellular-level visualization of the epidermis and superficial to mid-dermis by exploiting differences in refractive indices. Its advantages include high repeatability and real-time evaluation, making it valuable in the diagnosis of various skin disorders. Several studies have demonstrated the utility of RCM in identifying characteristic features of AK, thereby supporting its role in the diagnostic evaluation of these lesions [41,42].

RCM provides high-resolution, in vivo imaging of the skin, allowing detailed evaluation of morphological changes in AK. The RCM Vivascope 1500 enables imaging to a depth of 200–250 µm, sufficient to capture the full epidermis (with a thickness between 50 and 150 µm depending on the sun damage and site) and the superficial papillary dermis, which starts just below the dermal-epidermal junction (~100–150 µm) and extends to 200–250 µm. This range covers the anatomical layers typically involved in AKs, including the stratum corneum, spinous, basal, dermal-epidermal junction, and most of the papillary dermis where solar elastosis and inflammatory infiltrates are observed. Several RCM features, collectively referred to as RCM criteria, are particularly useful for identifying AK and differentiating it from other epidermal lesions [43,44]. One of the hallmark features is the abnormal honeycomb pattern in the spinous layer, reflecting disorganized keratinocyte architecture and disrupted intercellular cohesion. The disarranged spinous layer itself often exhibits nuclear polarization, where nuclei are aligned irregularly rather than in the orderly fashion of healthy epidermis [44,45,46]. Epidermal inflammatory infiltrate is frequently observed, consisting of small, bright, rounded cells indicative of inflammatory cell presence within the epidermis. In addition, dermal inflammatory infiltrate can be noted, highlighting the inflammatory response extending into the dermis [47].

Solar elastosis is another key criterion, representing degenerative changes in elastic fibers due to chronic UV exposure, often appearing as bright, tangled fibers in the papillary dermis. Dark central areas of parakeratosis correspond to retained nuclei within the stratum corneum, often seen as dense, dark aggregates surrounded by bright refractile structures. Detached corneocytes may also be present, appearing as loosely connected or free-floating cells within the superficial layers [44,45,46,47]. Additional criteria include round nucleated cells in the spinous layer, signifying dyskeratosis, and dendritic processes, which may reflect Langerhans cell activation or dendritic melanocytes. The presence of loss of honeycomb pattern—a disruption of the typical polygonal appearance of keratinocytes—further underscores the architectural disarray. Mottled pigmentation is characterized by uneven distribution of melanin or melanin-laden cells, while polycyclic papillary contours present as irregular, scalloped outlines of the dermal papillae. Small and bright papillae reflect changes in the papillary dermis, often corresponding to early neoplastic transformation [47,48,49].

Collectively, these RCM criteria provide a robust framework for the in vivo assessment of AK using RCM, enhancing diagnostic precision and potentially guiding management decisions.

## 3. Materials and Methods

### 3.1. Study Design and Ethical Aspects

A cross-sectional observational study was conducted at Elias Emergency University Hospital between April 2024 and July 2025 in order to determine the clinical, dermoscopic, and confocal microscopy scores for assessing the severity of AKs.

All participants provided written informed consent before enrollment. The approval of the Ethics Committee of the Elias Emergency University Hospital of Bucharest (Approval no. 1179/25 February 2025) was obtained. Patients’ rights were upheld according to the guidelines of the World Health Organization and the Helsinki Declaration.

### 3.2. Study Protocol

**Figure 1:**
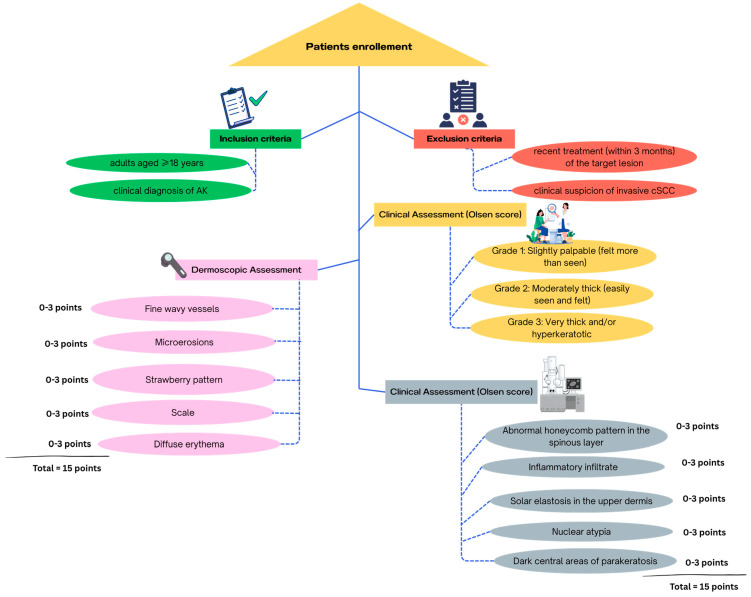
Study design and multimodal scoring system for AKs. Eligible patients met inclusion criteria and were assessed clinically (Olsen scale), dermoscopically, and by RCM. Both dermoscopic and RCM evaluations included five predefined features, each scored 0–3, for a maximum of 15 points per modality of evaluation. Image created in CanvaPro.

Fifty patients with fifty clinically diagnosed AKs were enrolled. The included patients were selected according with the inclusion and exclusion criteria presented in Figure 1.

**Figure 2:**
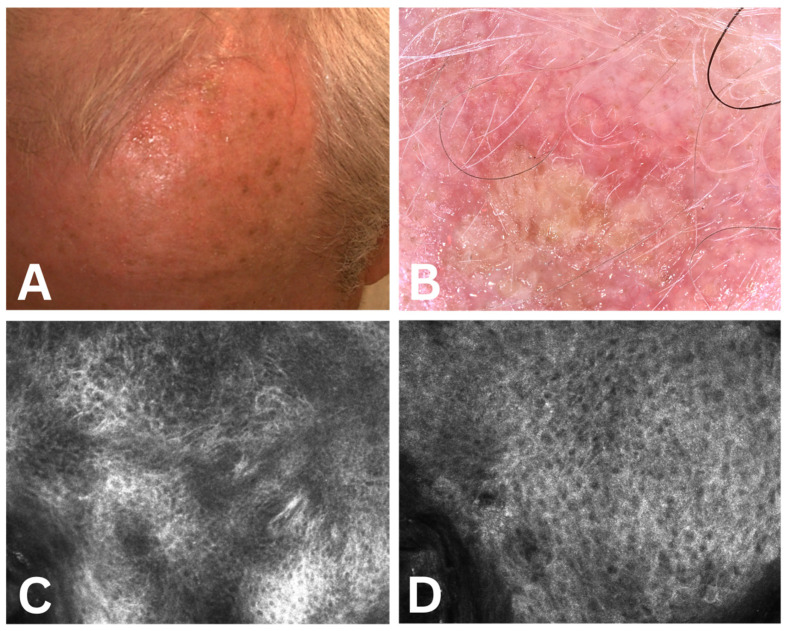
Case 1. (**A**) Actinic keratosis clinical aspect—Grade Olsen 2; (**B**) actinic keratosis dermoscopic score 7 (erosion—2 points, fine wavy vessels—2 points, strawberry pattern—1 point, erythema—1 point, scale—1 point); (**C**,**D**) actinic keratosis confocal microscopy score 4 points (abnormal honeycomb pattern 1 point, inflammation 1 point, elastosis 1 point, parakeratosis 1 point, cellylar atypia 0 points).

**Figure 3:**
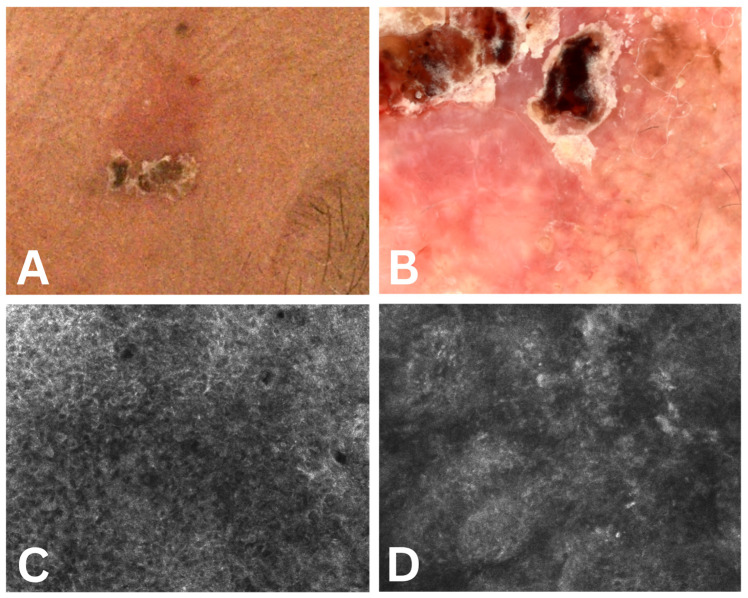
Case 2. (**A**) Actinic keratosis clinical aspect—Grade Olsen 2; (**B**) actinic keratosis dermoscopic score 6 (erosion—0 points, fine wavy vessels—3 points, strawberry pattern—1 point, erythema—1 point, scale—1 point); (**C**,**D**) actinic keratosis confocal microscopy score 5 points (abdnormal honeycomb pattern 2 points, inflammation 1 point, elastosis 2 points, parakeratosis 0 points, cellylar atypia 0 points).

**Figure 4:**
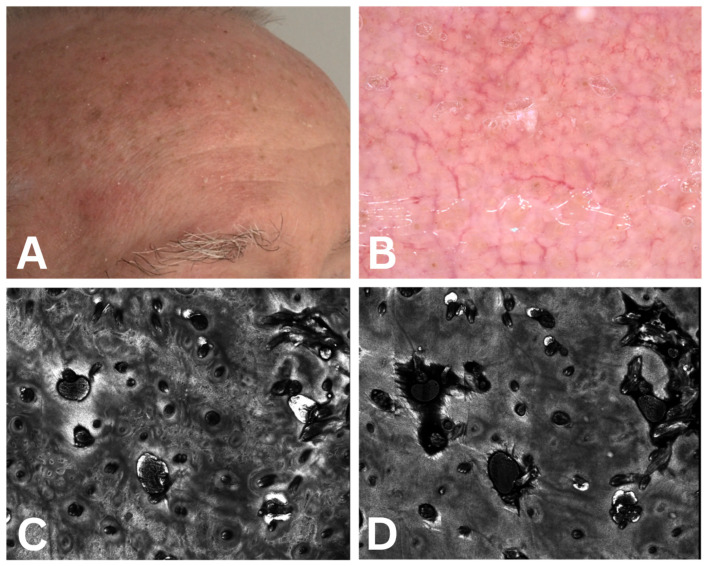
Case 3. (**A**) Actinic keratosis clinical aspect—Grade Olsen 3; (**B**) actinic keratosis dermoscopic score 11 (erosion—3 points, fine wavy vessels—1 point, strawberry pattern—1 point, erythema—3 points, scale—3 points); (**C**,**D**) actinic keratosis confocal microscopy score 10 points (abdnormal honeycomb pattern 3 points, inflammation 2 points, elastosis 2 points, parakeratosis 2 points, cellylar atypia 1 point).

The protocol for recording dermatoscopy and confocal microscopy images involved the following sequence of events: keeping the patient in an air-conditioned environment for 15 min prior to recording, clinical recording of the image, dermatoscopic, and subsequent RCM, according to the protocol developed by Guerra et al. [47]. The obtained images for three of the evaluated cases are presented in Figure 2, Figure 3 and Figure 4.

#### 3.2.1. Dermoscopic Assessment

For each patient we performed a dermoscopic evaluation, each lesion being evaluated for the presence and severity of fine wavy vessels, microerosions, strawberry pattern, scales, and diffuse erythema. Each feature was scored from 0 (absent) to 3 (severe). The total dermoscopy score ranged from 0 to 15.

#### 3.2.2. Confocal Microscopy Assessment

A semiquantitative scoring system was developed to evaluate actinic keratosis (AK) using in vivo reflectance confocal microscopy (RCM). This scoring system includes five RCM parameters commonly observed in AK such as, abnormal honeycomb pattern in the spinous layer, inflammatory infiltrate, solar elastosis in the upper dermis, nuclear atypia and dark central areas of parakeratosis, each graded on a scale from 0 to 3 based on severity. The cumulative score ranges from 0 to 15, allowing for stratification of lesion severity.

### 3.3. Data Analysis

Data analysis was performed using the IBM SPSS Statistics for Mac, Version 30.0 (released 2024; IBM Corp., Armonk, NY, USA). The chi-square test and Cramer’s V were used in order to establish the strength of association between evaluated variables. A *p*-value of <0.05 was considered significant.

## 4. Results

A total of 50 AK lesions from 50 patients (72% males, mean age = 69.76 years old 57–86 years, 62% Fitzpatrick phototype 3 and 38% Fitzpatrick phototype 2) were evaluated for clinical severity using the Olsen score, dermoscopic features, and confocal microscopy characteristics. Each lesion was assessed on a scale from 1 to 3, representing increasing clinical severity.

### 4.1. Association Between Clinical Severity and Dermoscopic Features

Dermoscopy features assessed included the presence and severity of fine wavy vessels, microerosions, strawberry pattern, scale, and diffuse erythema.

No statistically significant association was found between the Olsen clinical score and the degree of vascular structures (χ^2^(6) = 4.826, *p* = 0.566). The Cramér’s V value of 0.220 further confirms a weak association between the two variables. A statistically significant association was observed between the Olsen clinical severity score and the degree of erosions, as indicated by the Pearson chi-square test (χ^2^(6) = 21.387, *p* = 0.002). The strength of this association, as measured by Cramér’s V (0.462, *p* = 0.002), indicates a moderate relationship between clinical severity and the extent of erosions observed.

A statistically significant association was found between the Olsen clinical score and the presence of the strawberry pattern, as shown by the Pearson chi-square test (χ^2^(6) = 13.366, *p* = 0.038). This association indicates that the clinical severity of AK is moderately related to the appearance of the strawberry pattern. The Cramér’s V value of 0.366 suggests a moderate strength of association between clinical severity and the dermoscopic strawberry pattern. These findings suggest that the strawberry pattern may serve as a clinically relevant dermoscopic indicator of AK severity, although the relationship is moderate and not strictly linear.

A statistically significant and strong association was found between the Olsen clinical severity score and the degree of erythema, as indicated by the Pearson chi-square test (χ^2^(4) = 55.059, *p* < 0.001). The strength of this association was confirmed by a Cramér’s V value of 0.742, indicating a strong effect size. Additionally, a significant linear trend was observed (*p* < 0.001), suggesting that higher clinical severity scores were associated with increasing erythema intensity. These findings highlight erythema as a robust dermoscopic correlate of clinical severity in actinic keratosis.

A statistically significant association was observed between the Olsen clinical severity score and the degree of scaling, as demonstrated by the Pearson chi-square test (χ^2^(6) = 16.294, *p* = 0.012). The strength of this association, indicated by a Cramér’s V of 0.404, suggests a moderate correlation between the clinical stage of AK and the extent of hyperkeratotic surface features.

A statistically significant and strong association was identified between the Olsen clinical severity score and the total dermoscopic score, as demonstrated by the Pearson chi-square test (χ^2^(4) = 41.747, *p* < 0.001). The Cramér’s V value of 0.646 indicates a strong effect size, reflecting a robust relationship between clinical grading and cumulative dermoscopic findings. Additionally, the linear-by-linear association test (29.507, *p* < 0.001) supports a significant ordinal trend, suggesting that increases in the Olsen clinical score are consistently paralleled by higher dermoscopic composite scores.

### 4.2. Association Between Clinical Severity and Confocal Microscopy Features

A statistically significant and robust association was observed between the Olsen clinical severity score and the degree of abnormal honeycomb pattern detected by RCM, as indicated by the Pearson chi-square test (χ^2^(4) = 56.502, *p* < 0.001). The strength of this relationship is confirmed by a Cramér’s V of 0.752, denoting a strong effect size. Moreover, the linear-by-linear association was highly significant (33.832, *p* < 0.001), demonstrating a clear ordinal trend: as clinical severity increased (according to the Olsen score), the severity of honeycomb pattern disruption also intensified.

A statistically significant association was observed between the Olsen clinical severity score and the presence of nuclear atypia as detected by RCM, as shown by the Pearson chi-square test (χ^2^(2) = 9.049, *p* = 0.011). The association was further supported by a significant linear trend (*p* = 0.004), indicating that nuclear atypia becomes more prevalent as clinical severity increases. The effect size, as measured by Cramér’s V = 0.425, reflects a moderate strength of association. Biologically, this association reinforces the role of nuclear atypia as a key marker of dysplastic transformation in AK, reflecting progressive genomic instability and disrupted cell-cycle control. The observed correlation highlights the potential of in vivo RCM to detect early neoplastic changes, offering a valuable adjunct to clinical grading for stratifying lesion severity and risk of progression.

A statistically significant association was observed between the Olsen clinical severity score and the presence of inflammatory infiltrate as detected by RCM, as demonstrated by the Pearson chi-square test (χ^2^(4) = 14.771, *p* = 0.005). The Cramér’s V of 0.384 indicates a moderate effect size, suggesting a meaningful relationship between clinical severity and inflammatory cell presence. The linear-by-linear association test also reached statistical significance (*p* = 0.001), supporting an ordinal trend in which higher Olsen scores were associated with increased levels of epidermal or dermal inflammation.

A statistically significant association was found between the Olsen clinical severity score and the degree of solar elastosis detected by RCM, as demonstrated by the Pearson chi-square test (χ^2^(4) = 18.252, *p* = 0.001). The association showed a moderate strength, as indicated by Cramér’s V = 0.427. Furthermore, the linear-by-linear association test revealed a significant ordinal trend (*p* < 0.001), suggesting that higher Olsen scores are correlated with more advanced degrees of solar elastosis.

A statistically significant association was observed between the Olsen clinical severity score and the presence of parakeratosis as detected by RCM, as indicated by the Pearson chi-square test (χ^2^(4) = 15.142, *p* = 0.004). The Cramér’s V of 0.389 indicates a moderate effect size, suggesting a meaningful relationship between clinical severity and the degree of parakeratotic change. The linear-by-linear association test was also statistically significant (*p* < 0.001), indicating a positive ordinal trend: as clinical severity increases, parakeratosis becomes more prevalent or more severe.

A statistically significant and strong association was observed between the Olsen clinical severity score and the total RCM classification score, as demonstrated by the Pearson chi-square test (χ^2^(2) = 27.578, *p* < 0.001). The association exhibited a strong effect size, as indicated by Cramér’s V = 0.743. Additionally, a significant linear-by-linear trend was observed (*p* < 0.001), suggesting a clear ordinal relationship in which higher clinical severity (Olsen score) corresponds to more advanced RCM classification.

### 4.3. Association Between Overall Dermoscopic Score and Overall Confocal Microscopy Score

A statistically significant and strong association was found between the overall dermoscopic score and the overall RCM score, as indicated by the Pearson chi-square test (χ^2^(2) = 32.496, *p* < 0.001). The association demonstrated a very strong effect size, with Cramér’s V = 0.806 (*p* < 0.001). Furthermore, the linear-by-linear association test revealed a highly significant ordinal trend (*p* < 0.001), indicating that higher dermoscopic classification grades were consistently associated with higher RCM severity classifications.

### 4.4. Relationship Between Confocal Microscopy Features and Overall Dermoscopic Classification

The relationship between RCM features and overall dermoscopic score is depicted in Table 1.

A statistically significant and strong association was observed between the dermoscopic classification score and the severity of the abnormal honeycomb pattern detected by RCM, as demonstrated by the Pearson chi-square test (χ^2^(4) = 57.250, *p* < 0.001). The strength of the association is confirmed by a Cramér’s V of 0.757, indicating a strong effect size. Furthermore, the linear-by-linear test revealed a highly significant ordinal trend (*p* < 0.001), suggesting that higher dermoscopic severity scores are closely correlated with increasingly disrupted epidermal architecture, as visualized by the loss of the normal honeycomb pattern in RCM.

A statistically significant association was identified between the dermoscopic classification score and the presence of nuclear atypia detected by RCM, as indicated by the Pearson chi-square test (χ^2^(2) = 8.911, *p* = 0.012). The Cramér’s V of 0.422 (*p* = 0.012) denotes a moderate strength of association. Additionally, the linear-by-linear association test was statistically significant (*p* = 0.004), suggesting a positive ordinal trend: higher dermoscopic severity scores are associated with increased presence of nuclear atypia in confocal imaging.

A statistically significant association was found between the dermoscopic classification score and the intensity of inflammatory infiltrate detected by reflectance confocal microscopy, as indicated by the Pearson chi-square test (χ^2^(4) = 19.726, *p* < 0.001). The effect size was moderate to strong, with Cramér’s V = 0.444 (*p* < 0.001). The linear-by-linear association test also reached statistical significance (*p* < 0.001), indicating an ordinal relationship in which inflammatory cell presence increases with higher dermoscopic severity scores. This correlation highlights the biological coherence between surface features and cellular immune responses in AK.

A statistically significant association was observed between the dermoscopic classification score and the degree of solar elastosis identified by RCM, as shown by the Pearson chi-square test (χ^2^(4) = 20.613, *p* < 0.001). The effect size was moderate, with a Cramér’s V of 0.454 (*p* < 0.001). The linear-by-linear association test also reached significance (*p* < 0.001), indicating a strong ordinal relationship: higher dermoscopic classification scores were associated with increasing severity of solar elastosis on confocal imaging. From a biological standpoint, solar elastosis reflects chronic UV–induced damage to the dermal extracellular matrix, particularly degradation and accumulation of abnormal elastic fibers.

A statistically significant association was noticed between the dermoscopic classification score and the presence of parakeratosis identified by RCM, as shown by the Pearson chi-square test (χ^2^(4) = 26.466, *p* < 0.001). The strength of the association was moderate to strong, with a Cramér’s V of 0.514 (*p* < 0.001). The linear-by-linear association test also showed a highly significant ordinal trend (*p* < 0.001), indicating that increasing dermoscopic severity is associated with greater degrees of parakeratosis as detected on confocal microscopy.

These findings suggest that higher overall dermoscopic severity scores are consistently and significantly associated with the severity of these five confocal microscopy features. Among them, parakeratosis and abnormal honeycomb patterns show the strongest relationships, indicating their potential as confocal biomarkers of dermoscopic severity in AK.

### 4.5. Relationship Between Dermoscopic Features and Overall Confocal Microscopy Classification

The relationship between dermoscopic features and overall confocal microscopy score is depicted in Table 2.

There is no statistically significant association between the overall confocal classification and dermoscopic vascular score, the presence and aspect of fine wavy vessels. Although the Cramér’s V value suggests a weak association (0.272), this relationship lacks statistical significance (*p* = 0.296). This finding indicates that vascular patterns, as observed dermoscopically, may not reliably correlate with the confocal severity classification in this cohort, suggesting this feature may not meaningfully predict confocal severity.

Erosions showed a highly statistically significant association with overall confocal microscopy score (χ^2^(3) = 19.401, *p* < 0.001). The strength of the association was moderate to strong, as indicated by Cramér’s V (0.603). This suggests that erosions, although a dermoscopic feature, align well with increasing confocal severity, and likely reflect microscopic structural compromise in more advanced lesions. Erosions might be an indirect indicator of disrupted epidermal integrity, paralleling findings such as parakeratosis or loss of honeycomb architecture seen in RCM. 

No statistically significant association was found between the presence or intensity of the strawberry pattern on dermoscopy and the overall confocal microscopy score (χ^2^(3) = 5.609, *p* = 0.132), with Cramer’s V of 0.335 suggesting a weak relationship. Although the linear-by-linear association showed a weak trend (*p* = 0.036), this does not reach the typical threshold for significance after correction for multiple comparisons.

A statistically significant and strong association was observed between the dermoscopic erythema score and the overall confocal classification score, as indicated by the Pearson chi-square test (χ^2^(2) = 16.500, *p* < 0.001). The strength of this association was supported by a Cramér’s V value of 0.574 (*p* < 0.001), indicating a strong effect size. In addition, the linear-by-linear association test revealed a highly significant ordinal trend (*p* < 0.001), suggesting that increasing dermoscopic erythema severity scores were systematically associated with more advanced confocal classification stages.

Scale score was significantly associated with overall confocal microscopy score (χ^2^(3) = 20.061, *p* < 0.001). The Phi (0.633) and Cramer’s V (0.633) show a strong effect size, indicating that the presence of scale is related to confocal severity classification. Additionally, the linear-to-linear association test revealed a robust and statistically significant ordinal trend (*p* < 0.001), suggesting that increasing levels of dermoscopic scaling correlated with higher confocal classification severity. From a biologic standpoint, this relationship likely reflects the increasing hyperkeratosis and disrupted epidermal architecture observable on RCM in more advanced lesions.

Among these five features, erythema, scales, and microerosions demonstrated statistically significant association with confocal severity classification, while fine wavy vessels and strawberry pattern did not show significant relationships. 

### 4.6. Association Between Dermoscopy Severity Score, RCM Severity Score, and Sex

In our cohort of 50 patients diagnosed with AKs, 72% were male and 28% were female. This sex imbalance is consistent with previously reported epidemiological data, which show a higher prevalence of AK in male populations, likely due to increased cumulative lifetime sun exposure and lower photoprotective behaviors. While this predominance reflects the general epidemiology of AK, it could also influence our findings if sex-related differences exist in disease severity or treatment response. Therefore, analyses were stratified by sex to assess potential confounding effects.

The mean dermoscopy severity score was 6.19 +/− 2.606 in male patients and 5.50 +/− 1.743 in female patients. The Shapiro–Wilk test showed no violation of normality for either group (females—*p* = 0.196, males—*p* = 0.227), allowing for a parametric independent samples *t*-test. Levene’s test indicated equal variances (*p* = 0.117). The comparison revealed a slightly higher dermoscopic lesion severity in male patients, with no statistically significant difference, with a small size effect (Cohen’s d = −0.289). This suggests that sex did not influence the overall dermoscopic severity score in our cohort.

The mean RCM severity score was 5.44 +/− 2.36 in male patients and 5.00 +/− 1.617 in female patients. The Shapiro–Wilk test indicated that the overall RCM score was normally distributed in female patients (*p* = 0.066), but not in male patients (*p* = 0.003). Therefore, a non-parametric Mann–Whitney U test was used to compare scores between sexes. This showed a similar score distribution across groups, with mean ranks of 25.92 for males and 24.43 for females. The difference was not statistically significant (U = 237.0, Z = −0.382, *p* = 0.703), with a negligible effect size (r = 0.05). This suggests that sex did not influence the overall RCM severity score in this cohort.

## 5. Discussion

Statistical analyses revealed several key findings. First, a strong and significant association was observed between increasing clinical severity and multiple dermoscopic features, particularly erythema, scale, and erosions, all of which showed robust ordinal trends. Diffuse erythema emerged as the most faithful surrogate for clinical severity, reflecting the complex interplay between neoangiogenesis and chronic inflammation in ultraviolet-damaged skin. Microerosions, indicative of stratum-corneum fragility, also tracked with rising Olsen grades. In contrast, traditionally emphasized signs such as fine wavy vessels and the strawberry pattern, while diagnostically helpful, did not independently predict severity, underscoring the importance of feature weighting rather than checklist counting in dermoscopic algorithms. These surface-level characteristics were further validated by their significant correlation with RCM-derived parameters such as parakeratosis, nuclear atypia, inflammatory infiltrate, and abnormal honeycomb pattern—hallmarks of epithelial dysplasia and chronic UV-induced damage. The total dermoscopic score strongly correlated with both clinical and confocal severity scores, supporting its utility as a semi-quantitative, non-invasive surrogate marker for disease progression.

The high degree of concordance between overall dermoscopic and confocal classifications (Cramér’s V = 0.806, *p* < 0.001) underscores the interoperability of these modalities. This triangulation suggests that dermoscopy and RCM interrogate complementary strata of the same pathogenic spectrum: dermoscopy excels at depicting surface architecture and vascular tone, whereas RCM exposes the cellular-level chaos that heralds invasive transformation. Used in tandem, these modalities offer a holistic, layer-by-layer risk assessment that may obviate the need for routine punch biopsies across extensive field cancerization. This alignment supports the integration of structured dermoscopic and RCM-based scoring systems into multimodal diagnostic frameworks. From a translational perspective, this synergy enhances the non-invasive grading of AK and offers a reliable, reproducible foundation for lesion stratification, monitoring, and treatment selection.

Biologically, the findings affirm that surface-visible features—when rigorously assessed—serve as proxies for deeper epidermal and dermal pathology. This reinforces the conceptual framework of AK as a continuum of keratinocytic atypia and architectural disarray that begins with subtle histologic changes and progresses toward invasive transformation [50]. The integration of dermoscopy and RCM facilitates early identification of high-risk lesions, offering potential for pre-emptive intervention prior to malignant progression [43].

From a practical standpoint, our data support integrating structured dermoscopic and RCM scoring into outpatient workflows. Lesions exhibiting high erythema scores or elevated composite RCM scores could be prioritized for field-directed modalities such as topical 5-fluorouracil, photodynamic therapy, or ablative laser resurfacing, thereby reducing the therapeutic delay that often precedes progression to cSCC [51,52,53]. Conversely, low-score lesions might safely undergo active surveillance, minimizing overtreatment in elderly or polymorbid patients. The structured scores also provide an objective framework for monitoring treatment response and for comparing outcomes across clinical trials.

Nonetheless, by revealing robust, statistically significant linkages between clinical appearance, dermoscopic patterns, and confocal microarchitecture, this work lays the foundation for a unified, imaging-based severity index for AK. Such an index could harmonize eligibility criteria in interventional trials, facilitate personalized treatment algorithms, and serve as an early endpoint for chemopreventive strategies targeting field cancerization. Integration with artificial intelligence-driven image analysis may further enhance reproducibility and broaden access to advanced diagnostics beyond tertiary centers.

Diffuse dermoscopic erythema and key RCM hallmarks—disrupted honeycomb architecture, parakeratosis, nuclear atypia, inflammation, and solar elastosis—constitute high-impact, non-invasive biomarkers that dovetail with the Olsen grading system [12,38]. Their combined use promises to refine risk stratification, optimize therapeutic timing, and, most importantly, prevent the evolution of apparently innocuous keratinocytic dysplasias into life-threatening cSCC [54]. The present study represents a substantive step toward that goal.

While the present study was not designed to assess lesion progression, several of the features captured by our multimodal scoring system, particularly severe nuclear atypia, confluent parakeratosis, dense inflammatory infiltrate, and marked solar elastosis, have been associated in histopathological studies with higher-grade actinic keratosis and increased risk of progression to cSCC. Prior research has shown that AKs demonstrating loss of keratinocyte polarity, pronounced basal layer atypia, and extensive keratin retention often represent lesions at the upper end of the AK–SCC in situ continuum. Epidemiological studies estimate that while the annual per-lesion progression risk for AK is low (~0.6% at one year, ~2.6% at four years), lesions exhibiting multiple high-risk histological features carry a substantially greater likelihood of malignant transformation. Our scoring system was designed to non-invasively capture several of these high-risk morphologic traits. If validated in longitudinal cohorts, higher multimodal scores could serve not only for cross-sectional severity assessment but also for prognostic stratification, guiding more intensive surveillance or earlier intervention for lesions deemed at elevated risk [55,56,57,58].

## 6. Limitations

The present study is not without limitations. The modest sample size, single-center recruitment, and exclusion of immunocompromised individuals inevitably constrain external validity. Moreover, histopathological confirmation was reserved for atypical or therapy-resistant lesions, preventing a lesion-by-lesion comparison between RCM findings and invasive depth. Future multicenter studies with larger, histologically verified cohorts are essential to refine diagnostic cut-offs and to determine whether the proposed imaging thresholds predict longitudinal outcomes such as time to progression or recurrence after therapy.

## 7. Conclusions

This cross-sectional study provides one of the most comprehensive demonstrations to date that the macroscopic, dermoscopic, and cellular landscapes of AK describe a single, measurable continuum of disease severity. By prospectively correlating Olsen clinical grades with structured dermoscopic and RCM scores, we showed that non-invasive imaging not only mirrors bedside inspection but frequently offers a more nuanced view of early malignant potential. The approach confirms the long-held clinical intuition that AK is a field disease in which apparently heterogeneous lesions share a common biological trajectory toward cSCC.

In conclusion, this study supports the validity of combining clinical, dermoscopic, and confocal assessments in the grading of actinic keratosis. By elucidating statistically significant and biologically coherent associations among these modalities, it contributes to the development of a unified, non-invasive, and clinically applicable severity classification system. Such a framework has the potential to standardize lesion assessment, improve patient stratification, and ultimately guide therapeutic decision-making in the management of actinic keratosis and early cutaneous neoplasia.

## Figures and Tables

**Table 1:** Relationship between confocal microscopy features and overall dermoscopic score.

Score		Dermoscopy 1	Dermoscopy 2	Dermoscopy 3	*p*-Value
Abnormal honeycomb	0	0 (0%)	0 (0%)	0 (0%)	<0.001 **
	1	26 (89.65%)	3 (10.35%)	0 (0%)	
	2	1 (5.88%)	16 (94.12%)	0 (0%)	
	3	0 (0%)	2 (50%)	2 (50%)	
Nuclear atypia	0	25 (65.79%)	12 (31.58%)	1 (2.63%)	0.012 **
	1	2 (16.67%)	9 (75%)	1 (8.33%)	
	2	0 (0%)	0 (0%)	0 (0%)	
	3	0 (0%)	0 (0%)	0 (0%)	
Inflammation	0	1 (100%)	0 (0%)	0 (0%)	<0.001 **
	1	25 (73.53%)	8 (23.53%)	1 (2.94%)	
	2	1 (6.67%)	13 (86.67%)	1 (6.67%)	
	3	0 (0%)	0 (0%)	0 (0%)	
Elastosis	0	0 (0%)	0 (0%)	0 (0%)	<0.001 **
	1	24 (77.42%)	7 (22.58%)	0 (0%)	
	2	3 (18.75%)	11 (68.75%)	2 (12.5%)	
	3	0 (0%)	3 (100%)	0 (0%)	
Parakeratosis	0	14 (93.33%)	1 (6.67%)	0 (0%)	<0.001 **
	1	13 (48.15%)	14 (51.85%)	0 (0%)	
	2	0 (0%)	6 (75%)	2 (25%)	
	3	0 (0%)	0 (0%)	0 (0%)	
Overall RCM	1	26 (86.67%)	4 (13.33%)	0 (0%)	<0.001 **
	2	1 (5%)	17 (85%)	2 (10%)	
	3	0 (0%)	0 (0%)	0 (0%)	

** statistically significant; Dermoscopy 1 = Dermoscopy score between 0 and 5; Dermoscopy 2 = Dermoscopy score between 6 and 10; Dermoscopy 3 = Dermoscopy score between 11 and 15.

**Table 2:** Relationship between dermoscopic features and overall confocal microscopy score.

Score		MCR 1	MCR 2	MCR 3	*p*-Value
Vessels	0	3 (100%)	0 (0%)	0 (0%)	0.296
	1	15 (62.5%)	9 (37.5%)	0 (0%)	
	2	9 (47.37%)	10 (52.63%)	0 (0%)	
	3	3 (75%)	1 (25%)	0 (0%)	
Erosions	0	25 (78.12%)	7 (21.88%)	0 (0%)	<0.001 **
	1	5 (62.5%)	3 (37.5%)	0 (0%)	
	2	0 (0%)	7 (100%)	0 (0%)	
	3	0 (0%)	3 (100%)	0 (0%)	
Strawberry pattern	0	11 (84.62%)	2 (15.38%)	0 (0%)	0.132
	1	14 (53.85%)	12 (46.15%)	0 (0%)	
	2	5 (50%)	5 (50%)	0 (0%)	
	3	0 (0%)	3 (100%)	0 (0%)	
Erythema	0	0 (0%)	0 (0%)	0 (0%)	<0.001 **
	1	18 (90%)	2 (10%)	0 (0%)	
	2	12 (48%)	13 (52%)	0 (0%)	
	3	0 (0%)	5 (100%)	0 (0%)	
Scales	0	12 (100%)	0 (0%)	0 (0%)	<0.001 **
	1	15 (68.18%)	7 (31.82%)	0 (0%)	
	2	2 (22.22%)	7 (77.78%)	0 (0%)	
	3	1 (14.28%)	6 (85.72%)	0 (0%)	
Overall dermoscopy	1 (0–5)	26 (96.3%)	1 (3.7%)	0 (0%)	<0.001 **
	2 (6–10)	4 (19.05%)	17 (80.95%)	0 (0%)	
	3 (11–15)	0 (0%)	2 (100%)	0 (0%)	

** statistically significant; MCR 1 = Confocal microscopy score between 0 and 5; MCR 2 = Confocal microscopy score between 6 and 10; MCR 3 = Confocal microscopy score between 11 and 15.

## Abbreviations

The following abbreviations are used in this manuscript:

AK	actinic keratosis
RCM	reflectance confocal microscopy
OCT	optical coherence tomography
cSCC	cutaneous squamous cell carcinoma
